# Age-to-Glasgow Coma Scale score ratio predicts gastrointestinal bleeding in patients with primary intracerebral hemorrhage

**DOI:** 10.3389/fneur.2023.1034865

**Published:** 2023-02-13

**Authors:** Weizhi Qiu, Chubin Liu, Jinfu Ye, Gang Wang, Fuxing Yang, Zhigang Pan, Weipeng Hu, Hongzhi Gao

**Affiliations:** ^1^Department of Neurosurgery, The Second Affiliated Hospital of Fujian Medical University, Quanzhou, China; ^2^Department of Neurosurgery, The Second Affiliated Clinical Medical College of Fujian Medical University, Quanzhou, China; ^3^Department of Anesthesiology, The Second Hospital of Jinjiang, Quanzhou, China; ^4^Department of Neurosurgery, Lanzhou University Second Hospital, Lanzhou, China

**Keywords:** stress ulcer prophylaxis, gastrointestinal bleeding, predictors, stroke, prognosis, age, GCS

## Abstract

**Objective:**

Recent clinical studies have demonstrated that advanced age and low initial Glasgow Coma Scale (GCS) score were independent predictors of gastrointestinal bleeding (GIB) in patients with primary intracerebral hemorrhage (ICH). However, used singly, age and GCS score have their respective shortcomings in predicting the occurrence of GIB. This study aimed to investigate the association between the age-to-initial GCS score ratio (AGR) and the risk of GIB following ICH.

**Methods:**

We conducted a single-center, retrospective observational study of consecutive patients presenting with spontaneous primary ICH at our hospital from January 2017 through January 2021. Patients who fulfilled the inclusion and exclusion criteria were categorized into GIB and non-GIB groups. Univariate and multivariate logistic regression analyses were implemented to identify the independent risk factors for the occurrence of GIB, and a multicollinearity test was performed. Furthermore, one-to-one matching was conducted to balance important patient characteristics by the groups' propensity score matching (PSM) analysis.

**Results:**

A total of 786 consecutive patients fulfilled the inclusion/exclusion criteria for the study, and 64 (8.14%) patients experienced GIB after primary ICH. Univariate analysis revealed that patients with GIB were significantly older [64.0 (55.0–71.75) years vs. 57.0 (51.0–66.0) years, *p* = 0.001] and had a higher AGR [7.32 (5.24–8.96) vs. 5.40 (4.31–7.11), *p* < 0.001] and a lower initial GCS score [9.0 (7.0–11.0) vs. 11.0 (8.0–13.0)*, p* < 0.001]. The multicollinearity test revealed that no multicollinearity was observed in the multivariable models. Multivariate analysis showed that the AGR was a significant independent predictor of GIB [odds ratio (OR) 1.155, 95% confidence interval (CI) 1.041–1.281, *p* = 0.007], as well as prior anticoagulation or antiplatelet therapy (OR 0.388, 95% CI 0.160–0.940, *p* = 0.036) and MV used >24 h (OR 0.462, 95% CI 0.252–0.848, *p* = 0.013). Receiver operating curve (ROC) analysis illustrated that the optimal cutoff value for the AGR as a predictor for GIB in patients with primary ICH was 6.759 [the area under the curve (AUC) was 0.713 with a corresponding sensitivity of 60.94% and specificity of 70.5%, 95% CI 0.680–0.745, *p* < 0.001]. After 1:1 PSM, the matched GIB group had significantly higher AGR levels compared with the matched non-GIB group [7.47(5.38–9.32) vs. 5.24(4.24–6.40), *p* <0.001]. The ROC analysis indicated an AUC of 0.747 (the sensitivity was 65.62%, and the specificity was 75.0%, 95% CI 0.662–0.819, *p* < 0.001) for AGR levels as an independent predictor of GIB in patients with ICH. In addition, AGR levels were statistically correlated with unfunctional 90-day outcomes.

**Conclusion:**

A higher AGR was associated with an increased risk of GIB and unfunctional 90-day outcomes in patients with primary ICH.

## Introduction

Spontaneous primary intracerebral hemorrhage (ICH), a current significant disabling and killer disease worldwide, has the characteristics of high incidence, high prevalence, high mortality, high disability rate, and numerous lethal complications (1, 2). In addition to ICH severity, the complications of ICH are also associated with increased mortality and poor prognosis. Gastrointestinal bleeding (GIB) is a significant complication of acute ICH, which is significantly associated with an increased risk of mortality, length of stay in the intensive care unit (ICU), and unfavorable outcomes (3, 4). The reported incidence of GIB after ICH varies widely across studies, ranging from 16 to 54% (3, 5–8). However, previous reports in the literature have revealed that GIB is related to the morbidity of 48.3% and mortality as high as 87.9% following ICH (3, 5). Therefore, it is critical and crucial to identify people who are at high risk for GIB. Several indicators affecting GIB after acute ICH have been reported, including advanced age, lower Glasgow Coma Scale (GCS) score, ICH volume, and mechanical ventilation (MV) >48 h (3–5, 9). It is worth noting that there are consistent results from previous studies that the most relevant to the occurrence of GIB is neurological status and patient age (4, 5, 9, 10). Neurological status is typically measured using the GCS score, and clinicians assess the conscious state and brain functions through eye-opening, verbal, and motor responses. Age-related effects have been observed on ICH and GIB following ICH, including the initial physiological response (e.g., the presenting GCS), a particularly computed tomography (CT) finding, and the aggressiveness of neurosurgical management, morbidity, and mortality (5, 11).

The GCS score has the disadvantage that the summed score does not always accurately portray a patient's condition (12). It is now widely accepted that the GCS may be higher in the elderly than in younger patients for an equivalent anatomic severity of ICH. Despite near-normal GCS appearances, elderly patients may have severe anatomic ICH, with a high risk of subsequent GIB and poor prognosis (3, 5, 7). Accordingly, age may influence the relationship between the anatomic severity of ICH and neurological conditions measured by the GCS, ultimately affecting the prediction of GIB. Regrettably, most studies have not been adjusted for age and GCS score. To overcome the disadvantages of age and GCS score, we proposed a concept—age-to-GCS score ratio (AGR). The AGR was calculated by dividing age by the GCS score. In addition, a propensity score matching (PSM) analysis was conducted to reduce the potentially confounding elements affecting GIB in patients with ICH.

The present study aimed to investigate the predictive capacity of the AGR to identify GIB following ICH, and the AGR is expected to be a more practical tool for predicting GIB.

## Materials and methods

### Study population

Ethics approval for patient information was obtained from the Ethics Committee of the Second Affiliated Hospital of Fujian Medical University. We conducted a single-center, retrospective observational study of consecutive patients presenting with spontaneous primary ICH at our hospital from January 2017 through January 2021. Data were extracted from a prospectively maintained database containing demographic, clinical, operative, and follow-up data. The inclusion criteria were as follows: (1) age equal to or greater than 18 years; (2) an index ICH admission CT scan obtained within 24 hours (h) of symptom onset (for diagnostic confirmation and hematoma localization). Exclusion criteria were the following: (1) age less than 18 years old; (2) evidence of a secondary ICH etiology, including trauma, aneurysm, vascular malformation, moyamoya disease, hemorrhagic transformation of cerebral infarction, brain tumor, or any other cause of secondary ICH; (3) primary intraventricular hemorrhage (IVH); (4) active GIB (including esophageal and gastric variceal) on admission; (5) total gastrectomy; (6) known gastrointestinal (GI) lesions that might bleed (varies, polyps, and tumors); (7) peptic ulcer disease; and 8) historical modified Rankin scale (mRS) > 2.

All patients received the standard treatment according to the current ICH management guidelines (13). An indication for surgery was a midline shift more significant than 5 mm, a large hematoma <30 ml (supratentorial)/10 ml (infratentorial), or neurological impairment (14).

### Baseline data collection

Demographic variables included age and sex. Baseline characteristics related to medical history included the history of hypertension, diabetes mellitus, cigarette smoking, drug alcohol consumption, and prior anticoagulation or antiplatelet therapy as previously described (15, 16). Clinical neurological status on admission was evaluated with the GCS score, and if the patients were intubated and/or sedated, we used the best pre-intubation and post-resuscitation GCS scores. ICH characteristics recorded include the presence of intraventricular hemorrhage (IVH), ICH location, and hematoma volume. ICH hematoma volumes were determined on the initial CT using the ABC/2 method with an approximation of hematoma to an ellipsoid (17) and were categorized into large hematoma size (≥30 ml) or small hematoma size (<30 ml). In our clinical stroke database, ICH locations on admission CT were categorized as lobar (originating at the cortex and cortical–subcortical junction), deep (e.g., basal, ganglia, and thalami), cerebellar, or brainstem (18, 19). Peripheral venous blood was extracted per patient *via* venipuncture within 2 h of admission for laboratory examinations, including blood routine and blood coagulation. Blood coagulation included platelets, activated partial thromboplastin time (aPTT), international normalized ratio (INR), prothrombin time (PT), fibrinogen, and thrombin time. The patients invasively mechanically ventilated for more than 48 h were recorded. The age-to-GCS score ratio (AGR) was calculated by dividing age by the GCS score.

### Outcome assessments

The primary outcome was the occurrence of GIB within 14 days of acute ICH onset. GIB was defined as the presence of fresh blood or ground coffee in nasogastric aspirate, hematemesis, melena, blood in the stool, positive occult blood test, or positive fecal occult blood test during hospitalization (20). The patients received routine intravenous proton pump inhibitors (PPIs) during the hospital stay, 40 mg Q12H to treat GIB or 40 mg QD to prevent GIB (7).

The secondary outcome was functional independence (90-day, mRS 0–2). The functional outcome using the mRS score was assessed 90 days after the onset of ICH or until death, depending on which occurred first. All patients with ICH were followed up *via* outpatient records, telephone interviews, or WeChat (Tencent, Shenzhen, China) interviews. An mRS score of 0–2 was defined as a good prognosis, whereas an mRS of 3–6 was a poor prognosis.

### Statistical analysis

All statistical analyses were conducted with SPSS Statistics 25.0 software (SPSS Inc., Chicago, USA) and Prism 8.3.0 (GraphPad Software, San Diego, CA, USA). Prior to parametric statistical analysis, the data distributions were analyzed for normality using the Kolmogorov–Smirnov test (KS test). Baseline demographics and clinical characteristics were summarized as mean ± standard deviation (SD) for normally distributed continuous variables, median (interquartile range, IQR) for non-normally distributed continuous variables, and frequencies (percentages) for categorical variables. Student's t-test assessed comparisons between two groups for normally distributed variables and the Mann–Whitney U-test for non-normally distributed variables. In addition, comparisons were presented as violin plots with median and quartile values. Differences in categorical data were compared using the chi-square test or Fisher's exact test. Variables potentially associated with *p* < 0.1 in univariate analyses were included in the multivariate model. Multicollinearity was assessed using variance inflation factor (VIF) and tolerance before conducting the multivariable logistic regression analysis, and the model was subsequently adjusted to remove factors with obvious multicollinearity. Variables with VIF > 5 or tolerance < 0.2 indicated the existence of multiple collinearities removed from the model (21, 22). Receiver operating characteristic (ROC) curves were plotted along with the area under the ROC curve (AUC) using MedCalc (MedCalc Software, Ostend, Belgium). The ROC curves were used to determine the optimum cutoff points, whereby sensitivity and specificity were equally weighted. To further account for significant differences in baseline characteristics between non-GIB and GIB groups, we conducted a 1:1 propensity score matching (PSM) analysis. The variables with a *p* < 0.05 in the univariate analysis were incorporated into the PSM analysis. A two-sided *p* < 0.05 was considered statistically significant for all statistical analyses.

## Results

**Figure 1:**
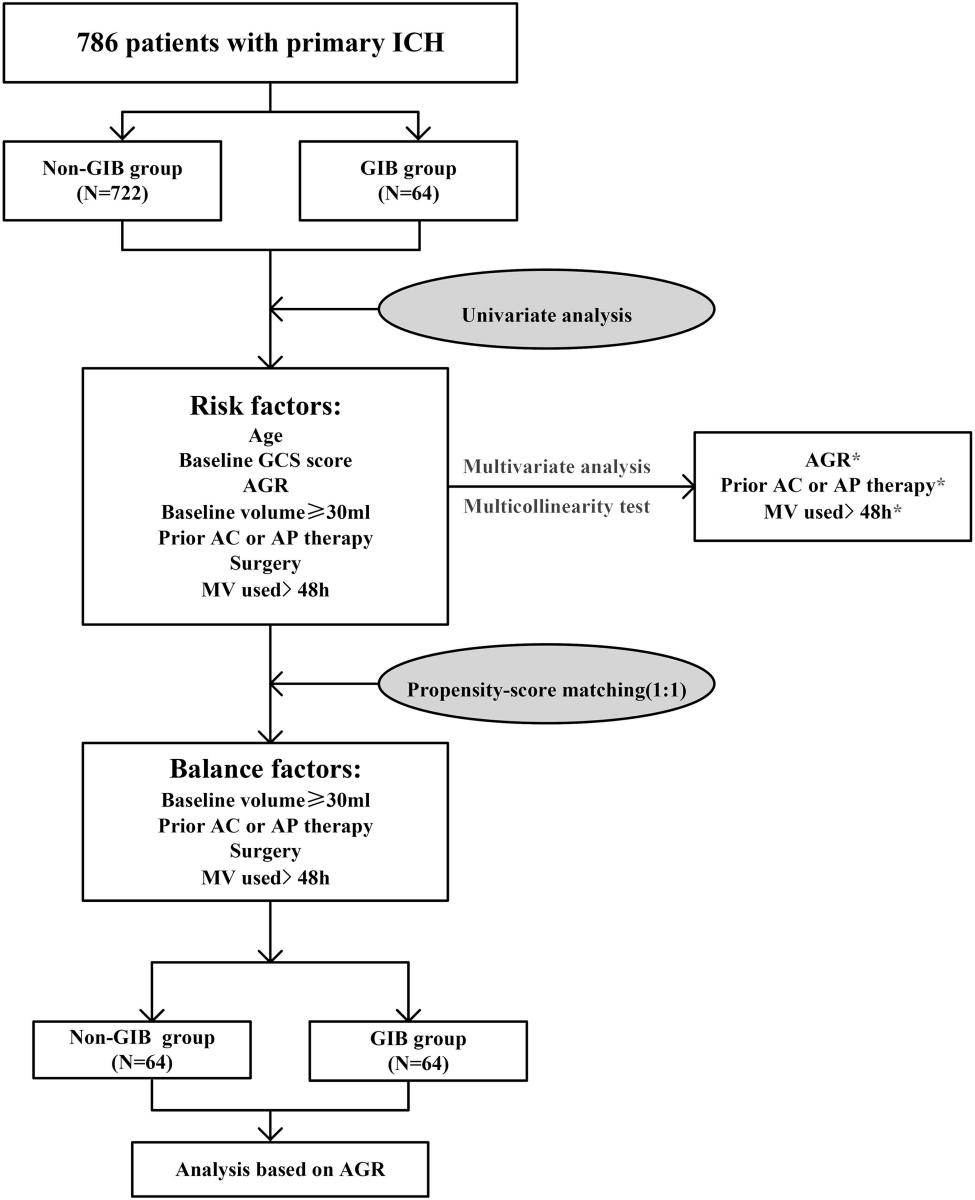
Study flow diagram and propensity score matching analysis process. AC, anticoagulation; AGR, age-to-GCS ratio; AP, antiplatelet; GCS, Glasgow Coma Scale; GIB, gastrointestinal bleeding; ICH, intracerebral hemorrhage; MV, mechanical ventilation. *p < 0.05.

Figure 1 illustrates the study flow diagram and PSM analysis process. A total of 786 consecutive patients fulfilled the inclusion/exclusion criteria for the study, and 64 (8.14%) patients experienced GIB after primary ICH. Baseline demographic and clinical characteristics are summarized in Table 1. The median age of included patients was 58 years (IQR, 52–66), and 497(63.2%) patients were male. The median time from illness onset to the first CT examination was approximately 4.0 h (IQR, 3.0–5.0 h), and the median GCS score upon admission was 12.0 (7.0–15.0). A total of 233 (29.6%) patients had an initial hematoma volume more significant than 30 ml.

**Table 1:** Patients' demographic and baseline characteristics.

**Characteristics**	**Values**
Number of patients	786
GI bleeding	64 (8.1)
Age, median (IQR), yrs	58 (52–66)
**Gender**	
Male (N, %)	497(63.2)
Female (N, %)	289 (36.8)
**Medical history**	
Smoking (N, %)	87 (11.1)
Alcohol (N, %)	71 (9.0)
Hypertension (N, %)	525 (66.8)
Diabetes mellitus (N, %)	117 (14.9)
Prior anticoagulation or antiplatelet therapy (N, %)	41 (5.2)
Time from symptom onset to initial CT, hours, median (IQR)	4.0 (3.0–5.0)
Baseline GCS score, median (IQR)	11.0 (8.0–13.0)
**Baseline volume (N, %)**	
< 30 ml	553 (70.4)
≥30 ml	233 (29.6)
Intraventricular hemorrhage (N, %)	200 (25.4)
**ICH location (n, %)**	
Deep	60.2 (76.6)
Lobar	114 (14.5)
Brain stem	29 (3.7)
Cerebellum	40 (5.1)
Hematoma expansion (N, %)	81 (10.3)
Age-to-GCS ratio, median (IQR)	5.40 (4.31–7.11)
Hemoglobin (g/L)	151.00 (138.0–163.0)
**Coagulation function, median (IQR)**	
Platelets (10^9^/L)	172.0 (136.0–212.25)
aPTT, s	24.80 (21.20–28.70)
INR	1.01 (0.94–1.07)
Prothrombin time, s	11.40 (10.80–12.0)
Fibrinogen, g/L	2.87 (2.40–3.40)
Thrombin time, s	17.30 (15.00–18.60)
MV used > 48 h (N, %)	128 (16.3)
Surgery (N, %)	353 (44.9)
Hospital length of stay, median (IQR), days	16.0 (10.0–23.0)
**Modified ranking scale (N, %)**	
0–2	328 (41.7)
3–6	458 (58.3)

aPTT, activated partial thromboplastin time; CT, computed tomography; GCS, Glasgow Coma Scale; ICH, intracerebral hemorrhage; INR, international normalized ratio; IQR, interquartile range; MV, mechanical ventilation.

**Figure 2:**
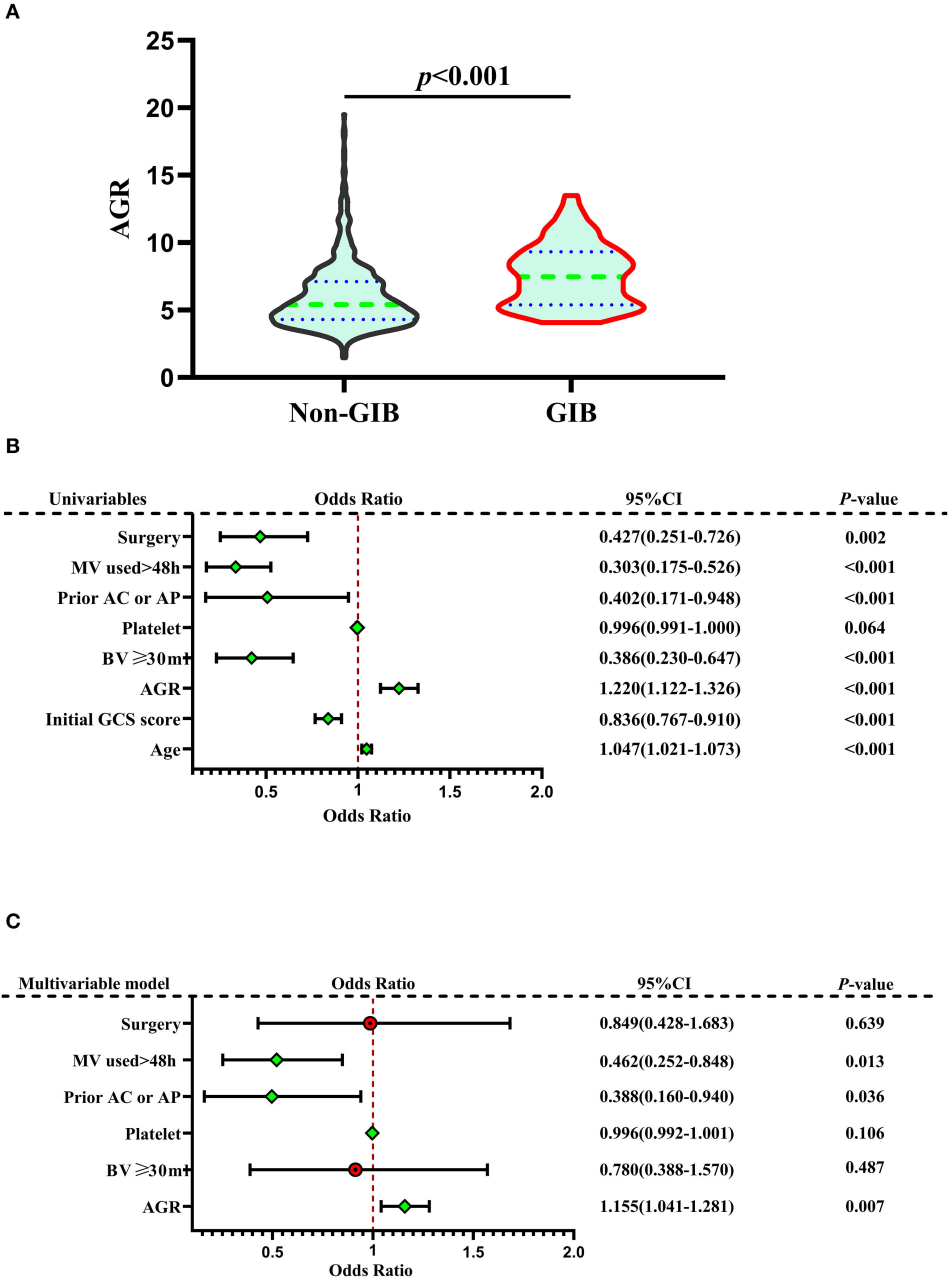
Correlation Between AGR and GIB. **(A)** Violin plot for comparing AGR between non-GIB and GIB groups. **(B)** Forest plots based on univariate analysis for risk factors associated with GIB. **(C)** Forest plots based on multivariate analysis for risk factors associated with GIB. In the multivariate logistic regression model, AGR level was a significant independent predictor of GIB, as well as prior anticoagulation or antiplatelet therapy and MV used >24 h. A median with an interquartile range was shown for the violin plot in **(A)**. Mann–Whitney U-tests were performed for comparison. AGR, age-to-GCS ratio; GCS, Glasgow Coma Scale; GIB, gastrointestinal bleeding; ICH, intracerebral hemorrhage; MV, mechanical ventilation; Prior AC or AP, prior anticoagulation or antiplatelet therapy.

**Figure 3:**
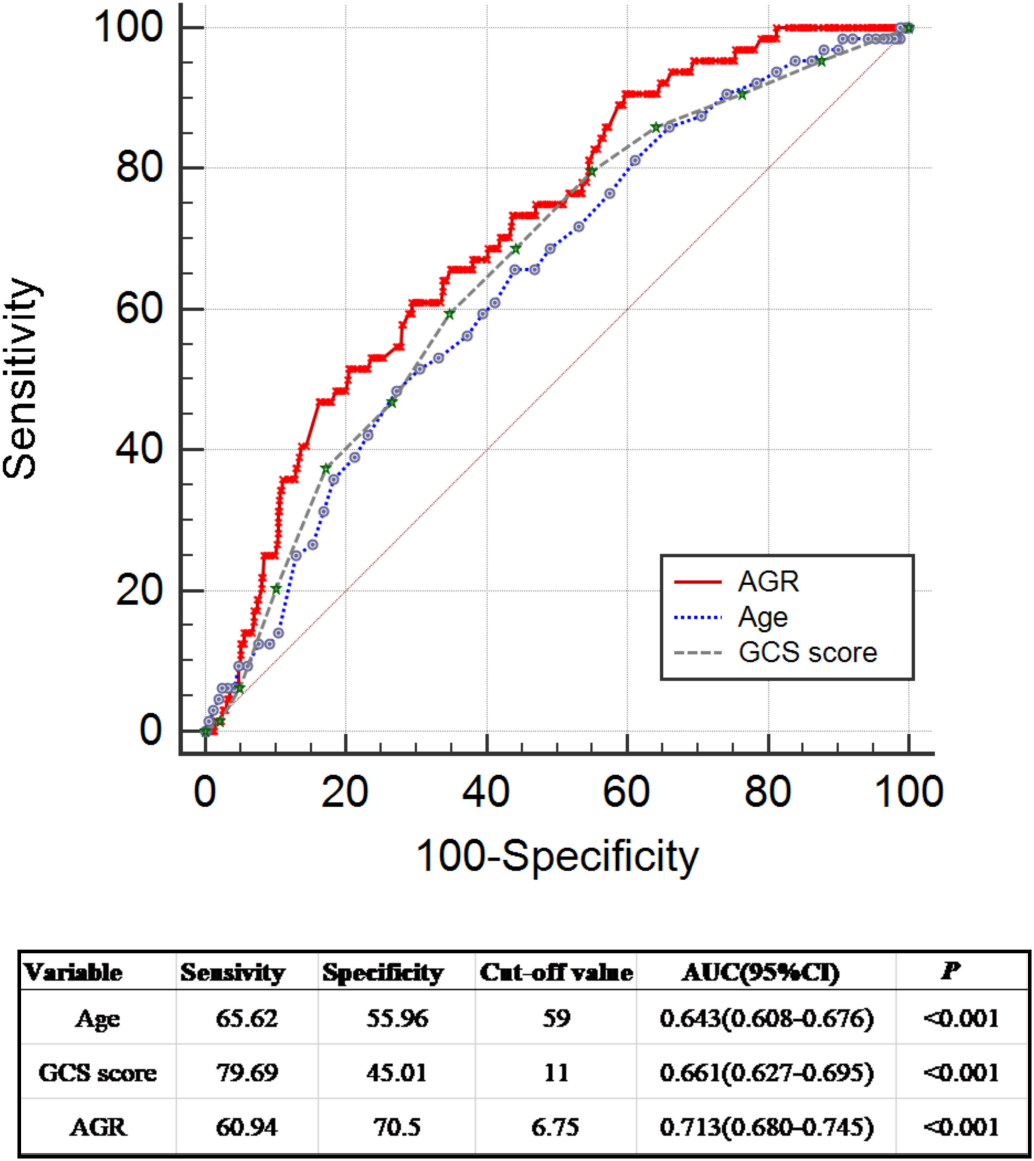
Receiver operating curve analyses comparing AGR, age, and initial GCS score for predicting GIB in patients with ICH. ROC curves were plotted for AGR, age, and initial GCS score, with an AUC of 0.713 (95% CI 0.680–0.745, *p* < 0.001), 0.643 (95% CI 0.608–0.676, *p* < 0.001), and 0.661 (95% CI 0.627–0.695, *p* < 0.001), respectively. Z-test demonstrated that the AUC of AGR and the AUCs of age and initial GCS score were comparable (AGR vs. age: Z = 1.798, *p* = 0.0722; AGR vs. GCS score: Z = 2.865, *p* = 0.0042; age vs. GCS score: Z = 0.351, *p* = 0.7256). The AUC of AGR was significantly higher than the AUC of GCS score (AGR vs. GCS score: Z = 2.865, *p* = 0.0042). The AUC of age was slightly lower than that of AGR without statistical significance (Z = 1.798, *p* = 0.0722). AGR, age-to-GCS ratio; AUC, area under the curve; CI, confidence interval; GCS, Glasgow Coma Scale; GIB, gastrointestinal bleeding; ICH, intracerebral hemorrhage; ROC, receiver operating curve.

Seven hundred eighty-six patients with primary ICH were stratified into two groups: absence (non-GIB group, *N* = 722) or presence of GIB (GIB group, *N* = 64). Univariate analysis revealed significant differences between the two groups in terms of age, baseline GCS score, baseline volume (BV) ≥ 30 ml, prior anticoagulation or antiplatelet therapy, mechanical ventilation (MV) used >24 h, surgery, and AGR. When compared with patients without GIB, those with GIB were significantly older [64.0 (55.0–71.75) years vs. 57.0 (51.0–66.0) years, *p* = 0.001] and had a higher AGR [7.32 (5.24–8.96) vs. 5.40 (4.31–7.11), *p* < 0.001] and a lower initial GCS score [9.0 (7.0–11.0) vs. 11.0 (8.0–13.0)*, p* < 0.001] (Table 2, Figures 2A, B). The AGR was positively associated with the occurrence of GIB with an odds ratio (OR) value of 1.220 [95% confidence interval (CI) 1.122–1.326, *p* < 0.001] in the unadjusted model (Figure 2B). Multicollinearity analyses of the included variables were undertaken, and no multicollinearity was observed in the multivariable models as judged by VIF and tolerance (Table 3). We, therefore, carried out a stepwise multivariate analysis incorporating six covariates (Figure 2C). The multivariate analysis results were adjusted for the following confounding factors: platelet, BV ≥ 30 ml, prior anticoagulation or antiplatelet therapy, surgery, and MV used >24 h. After adjustment in the multivariate logistic regression model, AGR level was a significant independent predictor of GIB (OR 1.155, 95% CI 1.041–1.281, *p* = 0.007), as well as prior anticoagulation or antiplatelet therapy (OR 0.388, 95% CI 0.160–0.940, *p* = 0.036) and MV used >24 h (OR 0.462, 95% CI 0.252–0.848, *p* = 0.013) (Figure 2C). However, BV ≥ 30 ml, platelet, and surgery were not independent predictors of GIB (*p* > 0.05; Figure 2C)_._ The Hosmer–Lemeshow test was applied to assess goodness-of-fit for the multivariable logistic regression model, indicating that the model was an appropriate fit (χ^2^ = 11.336, *p* = 0.183). The resulting ROC curves and associated AUC values of age, GCS score, and AGR are visualized in Figure 3. The optimal cutoff value for AGR as a predictor for GIB in patients with primary ICH was 6.759(the AUC was 0.713 with a corresponding sensitivity of 60.94% and specificity of 70.5%, 95% CI 0.680–0.745, *p* < 0.001; Figure 3). The AUC of the AGR level and the GCS score were comparable by Z-test (*p* = 0.0042), and the AUC of AGR was statistically higher than that of the GCS score.

**Table 2:** Characteristics of all patients with or without gastrointestinal bleeding.

**Characteristics**	**non-GIB group** **(*N* = 722)**	**GIB group** **(*n* = 64)**	**Univariate** ***P* value**
Age, median (IQR), yrs	57 (51–66)	64 (55–71.75)	0.001
**Gender**			0.866
Male (N, %)	456 (63.2)	41 (64.1)	
Female (N, %)	266 (36.8)	23 (35.9)	
Medical history			
Smoking (N, %)	80 (11.1)	7 (10.9)	0.972
Alcohol (N, %)	63 (8.7)	8 (12.5)	0.313
Hypertension (N, %)	477 (66.1)	48 (75.0)	0.146
Diabetes mellitus (N, %)	104 (14.4)	13 (20.3)	0.203
Prior anticoagulation or antiplatelet therapy (N, %)	34 (4.7)	7 (10.9)	0.032
Time from symptom onset to initial CT, hours, median (IQR)	4.0 (3.0–5.0)	4.0 (3.0–5.0)	0.542
Baseline GCS score, median (IQR)	11.0 (8.0–13.0)	9.0 (7.0–11.0)	< 0.001
**Baseline volume (N, %)**			< 0.001
< 30 ml	521 (72.2)	32 (50.0)	
≥30 ml	201 (27.8)	32 (50.0)	
**ICH location (n, %)**			0.370
Deep	500 (76.3)	52 (81.3)	
Lobar	106 (14.7)	8 (12.5)	
Brain stem	29 (4.0)	0 (0.0)	
Cerebellum	36 (5.0)	4 (6.3)	
Intraventricular hemorrhage (N, %)	187 (25.9)	13 (20.3)	0.325
Hemoglobin (g/L)	151.0 (139.0–163.0)	148.0 (136.0–169.0)	0.517
**Coagulation function, median (IQR)**			
Platelets (10^9^/L)	173.0 (136.0–215.0)	162.50 (129.25–190.25)	0.067
aPTT, s	24.7 (21.30–28.70)	25.70 (22.0–30.70)	0.200
INR	1.01 (0.94–1.07)	1.03 (0.95–1.09)	0.159
Prothrombin time, s	11.40 (10.80–12.0)	11.45 (10.90–12.0)	0.657
Fibrinogen, g/L	2.85 (2.40–3.40)	2.90 (2.27–3.47)	0.609
Thrombin time, s	17.40 (15.10–18.60)	17.0 (14.60–18.90)	0.363
MV used >48 h (N, %)	105 (14.5)	23 (35.9)	< 0.001
Surgery (N, %)	312 (43.2)	41 (64.1)	0.001
AGR, median (IQR)	5.40 (4.31–7.11)	7.47 (5.38–9.32)	< 0.001
**Modified Rankin Scale (N, %)**			0.005
0–2	312 (43.2%)	16 (25.0)	
3–6	410 (56.8%)	48 (75.0)	

AGR, age-to-GCS ratio; aPTT, activated partial thromboplastin time; CT, computed tomography; GCS, Glasgow Coma Scale; ICH, intracerebral hemorrhage; INR, international normalized ratio; IQR, interquartile range; MV, mechanical ventilation.

**Table 3:** Multicollinearity test for the factors of a multivariate model.

**Multicollinearity statistics**		
**Independent variable**	**Tolerance**	**Variable inflation factor (VIF)**
AGR	0.714	1.401
Prior anticoagulation or antiplatelet therapy	0.996	1.004
Baseline volume ≥30 ml	0.583	1.716
Platelet	0.992	1.008
MV used > 24 hours	0.859	1.164
Surgery	0.646	1.548

AGR, age-to-GCS ratio; MV, mechanical ventilation.

**Figure 4:**
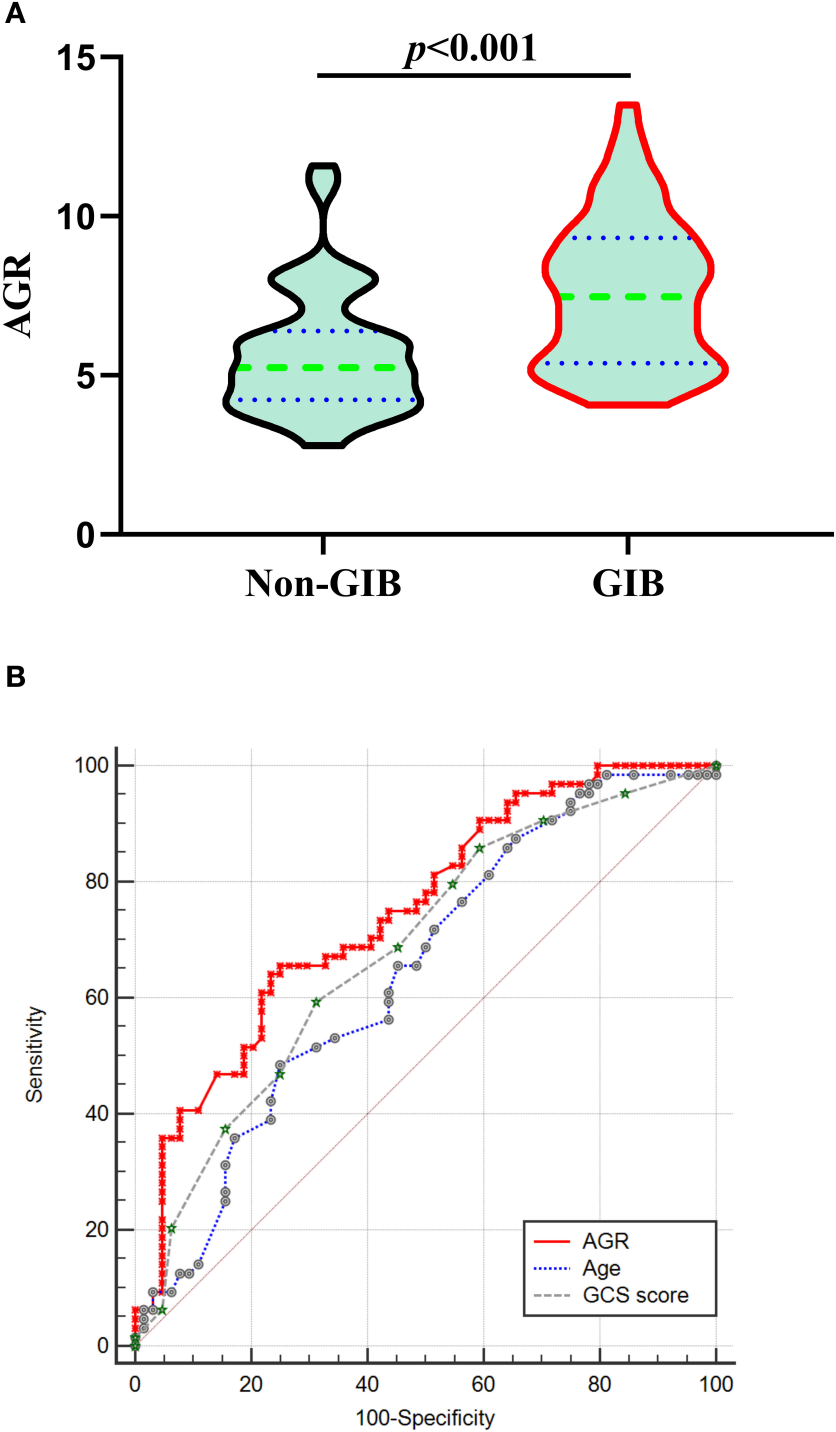
Correlation Between AGR and GIB after PSM. **(A)** Violin plot for comparing AGR using Mann-Whitney tests between non-GIB and GIB group after PSM. **(B)** After PSM, ROC curves were plotted for AGR, age and initial GCS score, with an AUC of 0.747 (95%CI 0.662–0.819, *p* < 0.001), 0.646 (95%CI 0.557–0.728, *p* = 0.0027), and 0.682 (95%CI 0.594–0.762, *p* < 0.001), respectively. AGR: age-GCS ratio; AUC, area under the curve; CI: confidence interval; GCS: Glasgow Coma Scale; GIB: gastrointestinal bleeding; ICH, intracerebral hemorrhage; PSM: propensity-score matching; ROC, Receiver operating curve.

We conducted a 1:1 PSM analysis to match patients without GIB with patients with GIB, balancing the differences in the baseline characteristic. The significantly different in BV ≥ 30 ml, prior anticoagulation or antiplatelet therapy, MV used >24 h, and surgery between the two groups was balanced. The PSM analysis identified 64 pairs of patients, with 64 patients in each group. The characteristics of 128 matched patients based on the PSM (64 in each group) are summarized in Table 4. The matched GIB group had significantly higher AGR levels compared with the matched non-GIB group [7.47 (5.38–9.32) vs. 5.24(4.24–6.40), *p* < 0.001; Table 4, Figure 4A] in the univariate analysis (Figure 4A). After PSM, ROC analysis indicated an AUC of 0.747(the sensitivity was 65.62%, and the specificity was 75.0%, 95% CI 0.662–0.819, *p* < 0.001) for AGR levels as an independent predictor of GIB in patients with ICH (Figure 4B). The higher AGR level was still an independent predictor of GIB. Interestingly, a significant difference was witnessed between the ICH location and GIB after PSM (Table 4).

**Table 4:** Characteristics of all patients with or without gastrointestinal bleeding after PSM.

**Characteristics**	**Non-GI bleeding** **(*N* = 64)**	**GI bleeding** **(*N* = 64)**	**Univariate** ***P* value**
Age, median (IQR), yrs	57.5 (49.5–65.75)	65.0 (56.0–71.75)	0.004
**Gender**			1.000
Male (N, %)	41 (64.1)	41 (64.1)	
Female (N, %)	23 (35.9)	23 (35.9)	
**Medical history**			
Smoking (N, %)	4 (6.3)	7 (10.9)	0.344
Alcohol (N, %)	5 (7.8)	8 (12.5)	0.388
Hypertension (N, %)	41 (64.1)	48 (75.0)	0.179
Diabetes mellitus (N, %)	9 (14.1)	13 (20.3)	0.349
Prior anticoagulation or antiplatelet therapy (N, %)	13 (20.3)	7 (10.9)	0.144
Time from symptom onset to initial CT, hours, median (IQR)	4.0 (3.0–5.0)	4.0 (3.0–5.0)	0.296
Baseline GCS score, median (IQR)	11.0 (8.25–14.0)	9.0 (7.0–11.0)	< 0.001
**Baseline volume (N, %)**			0.375
< 30 ml	37 (57.8)	32 (50.0)	
≥30 ml	27 (42.2)	32 (50.0)	
**ICH location (N, %)**			0.004
Deep	34 (53.1)	52 (81.3)	
Lobar	22 (34.4)	8 (12.5)	
Brain stem	3 (4.7)	0 (0.0)	
Cerebellum	5 (7.8)	4 (6.3)	
Intraventricular hemorrhage (N, %)	13 (20.3)	13 (20.3)	1.000
Hemoglobin (g/L)	150.0 (141.0–164.0)	148.0 (136.0–169.0)	0.625
**Coagulation function, median (IQR)**			
Platelets (10^9^/L)	173.50 (142.00–201.75)	162.50 (129.25–190.25)	0.287
aPTT, s	26.35 (22.33–30.03)	25.70 (22.00–30.70)	0.786
INR	1.02 (0.97–1.02)	1.03 (0.95–1.09)	0.784
Prothrombin time, s	11.70 (11.00–12.20)	11.45 (10.90–12.00)	0.289
Fibrinogen, g/L	2.78 (2.30–3.49)	2.90 (2.27–3.47)	0.545
Thrombin time, s	17.35 (15.13–18.70)	17.0 (14.60–18.90)	0.324
MV used >48 h (N, %)	22 (34.4)	23 (35.9)	0.853
Surgery (N, %)	36 (56.3)	41 (64.1)	0.367
AGR, median (IQR)	5.24 (4.24–6.40)	7.47 (5.38–9.32)	< 0.001
**Modified Rankin Scale (N, %)**			< 0.001
0–2	47 (73.4)	16 (25.0)	
3–6	17 (26.6)	48 (75.0)	

AGR, age-to-GCS ratio; aPTT, activated partial thromboplastin time; CT, computed tomography; GCS, Glasgow Coma Scale; ICH, intracerebral hemorrhage; INR, international normalized ratio; IQR, interquartile range; PSM, propensity score matching.

**Figure 5:**
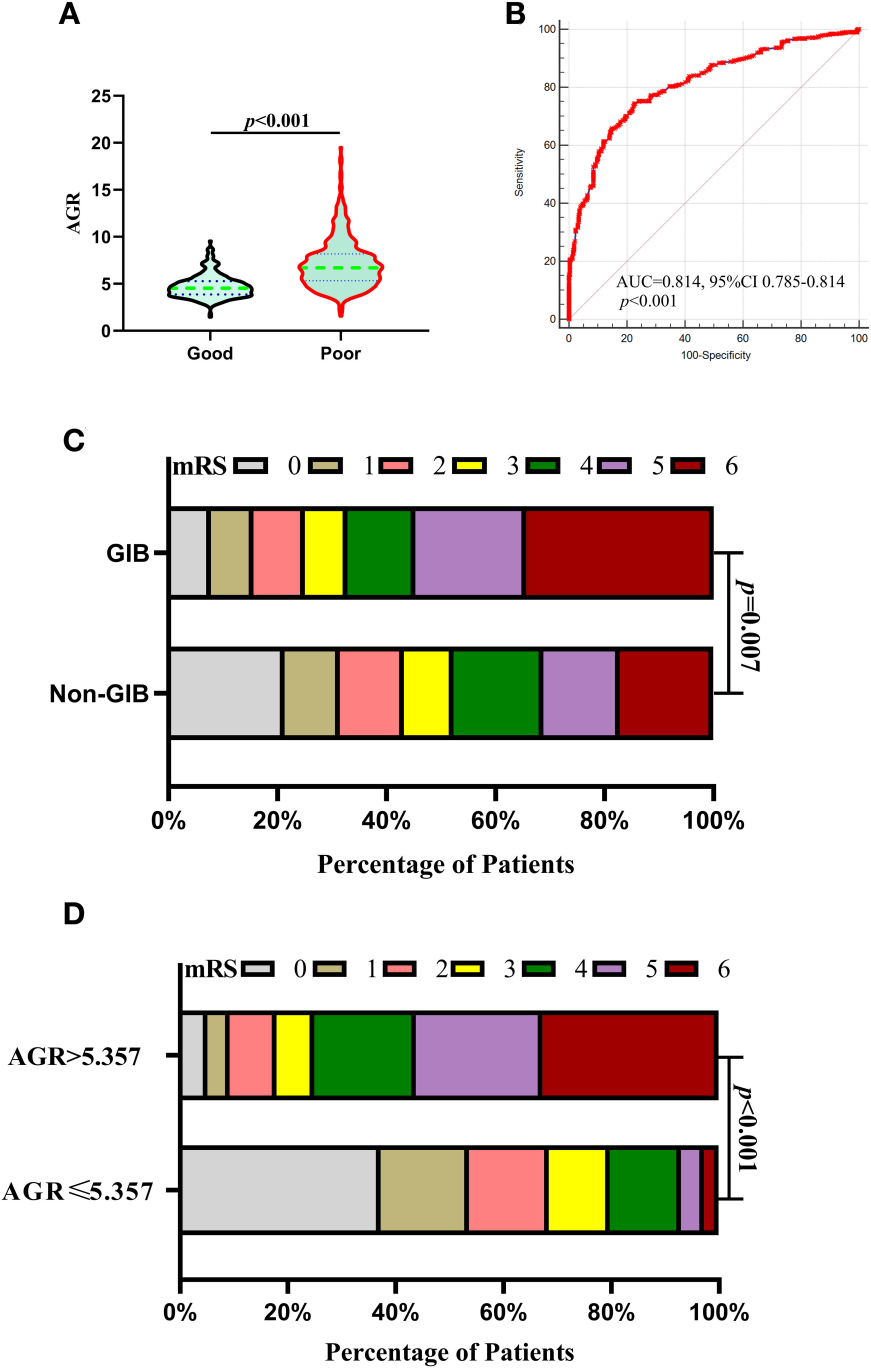
Association of AGR with outcomes after 1:1 PSM analysis. **(A)** Violin plot for comparing AGR using Mann–Whitney U-tests between good and poor outcomes after PSM. **(B)** ROC analysis illustrated the predictive power of AGR (the AUC was 0.814, 95% CI 0.785 to 0.8411, *p* < 0.0001; the sensitivity was 74.45%, and the specificity was 77.44%) for the outcome after PSM. **(C)** Distributions of mRS scores at 90 days between the GIB and non-GIB groups. A statistical difference was found between the two groups. **(D)** Functional outcome at 90 days for patients with lower AGR (AGR > 5.357) and higher AGR (AGR ≤ 5.357). A statistically significant difference was found in the comparison between the two groups. **(C, D)** Proportions of patients within each score category on the 7-point scale (where 0 indicates no symptoms and 6 indicates death) at 90 days after illness onset. AGR, age-to-GCS ratio; AUC, area under the curve; CI, confidence interval; GCS, Glasgow Coma Scale; GIB, gastrointestinal bleeding; ICH, intracerebral hemorrhage; PSM, propensity score matching; ROC, Receiver operating curve.

Patients in the poor prognosis group had a significantly higher AGR level than those in the good prognosis group (Figure 5A). The ROC analysis illustrated the predictive power of AGR (the AUC was 0.814, 95% CI 0.785 to 0.8411, *p* < 0.0001; the sensitivity was 74.45%, and the specificity was 77.44%) for the outcome (Figure 5B), indicating that AGR was a prognostic predictor in patients with ICH. Patients with GIB had a statistically worse prognosis than those without GIB (Tables 2, 4, Figure 5C), either before or after PSM. Figure 5C depicts the 90-day mRS after illness onset for patients with GIB and without GBI. Patients with an AGR > 5.357 had a statistically worse prognosis than patients with an AGR ≤5.357. The distribution of mRS scores is demonstrated in Figure 5D for the two groups of patients, and a statistically significant difference was observed.

## Discussion

In the present study, we identified that a higher AGR level was an independent predictor of GIB following primary ICH. Furthermore, AGR was a statistically better predictor than the GCS score, a well-established predictor in predicting GIB. Even in the patients with selected PSM adjusted for the differences in BV ≥ 30 ml, prior anticoagulation or antiplatelet therapy, mechanical ventilation (MV) used >24 h, and surgery (they were balanced in PSM), our study demonstrated a similar conclusion that the higher AGR was still an independent predictor of GIB in patients with ICH. We additionally observed that AGR levels were statistically correlated with unfunctional 90-day outcomes. To the best of our knowledge, this is the first study to propose the concept of AGR and report the potential predictive power of elevated AGR for GIB in patients with ICH.

GIB is a severe complication of acute ICH. Identified risk factors for GIB may help clinicians identify the risks of GIB before it develops. In accordance with previous reports, age was an independent predisposing factor for GIB, with a markedly increased risk after age 65 (23) and even more so in ICH. A retrospective review of 808 ICH cases demonstrated that elderly patients with ICH significantly increased the risk for GIB (5). A study by Chen et al. found that 20.5% of elderly patients with stroke developed GIB (24). Similarly, accumulating studies have confirmed that advanced age is an independent predictor of GIB occurring after ICH (24–26). Elderly patients with ICH, especially those with other comorbidities (e.g., infection, renal insufficiency, and replacement therapy) (5–7, 9), are more likely to develop GIB. Furthermore, disruption of the axis between the central nervous system and the gastrointestinal system may lead to gastrointestinal bleeding or dyskinesia, which may be more pronounced in elderly patients with ICH (23, 24, 27). Third, an early study documented that hyperactivity of the vagus nerve after brain injury, including ICH, may induce gastric acid hypersecretion and gastric mucosal damage, ultimately leading to GIB (28). This condition is more common in elderly patients with cerebral hemorrhage.

Numerous studies have previously confirmed that a lower GCS score is statically associated with GIB after ICH. However, the underlying mechanism is less understood. The GCS score has been used to assess the severity of neurological deficits (29), focusing on vital functions of the central nervous system, including eye-opening, language, and motor responses. The GCS score correlates with the level of consciousness. Thus, a decreased GCS score in the acute phase of ICH is associated with neurological impairment. A recent study has reported that impaired consciousness or conscious disturbance (GCS score <8) was the most critical risk factor associated with GIB in patients with stroke (24). People with impaired consciousness are more likely to experience breaks in the axis between the digestive and nervous systems than those with clear consciousness (24, 27), resulting in GIB.

Although multiple factors contribute to the development of GIB, ischemia and reperfusion injury are the primary pathophysiological mechanism leading to GIB (7). The acute rise in intracranial pressure (ICP) associated with ICH may result in vagal hyperactivity and increased gastric acid secretion (8). A larger hematoma may increase ICP when ICH occurs. A larger initial intracranial hematoma occurs, followed by an increase in ICP and a decrease in GCS score. A decreased GCS score may be one of the clinical manifestations of elevated ICP. Prior literature has demonstrated that the occurrence of GIB is significantly associated with surrogate markers of increased ICP (8, 30). In the present study, decreased initial GCS score was observed in the GIB group after ICH, supporting a possible link between GCS score, ICP, and GIB. We speculated that the mechanisms were as follows. First, the raised ICP may cause vagal hyperactivity, leading subsequently to mucosal ischemia and increased gastric acid secretion, resulting in GIB. Second, a sharp increase in ICP may lead to excessive cholinergic activity, which increases gastric acid production. Third, elevated catecholamine concentrations in patients with ICH may cause vasoconstriction and ulceration of the gastrointestinal mucosa, ultimately leading to gastric bleeding (24). Fourth, disruption of the axis between the central nervous system and the digestive system due to severe stroke (GCS score <8) may increase the risk of mucosal injury in the digestive system (23, 24). Finally, following ICH stress, especially severe ICH (GCS score <8), reactive oxygen metabolites and various proinflammatory mediators increase (18, 31). Alternation in these proinflammatory mediators, neutrophils, and mast cells may all potentially contribute to reperfusion-related gastric injury (8).

Our study verifies that an elevated AGR level can predict GIB following ICH. After multivariate and PSM analyses, AGR remained a valuable predictor for GIB following ICH. The ROC curve illustrated that the AUC of AGR was significantly higher than the GCS score (AGR vs. GCS score: Z = 2.865, *p* = 0.0042). The AUC of AGR was slightly higher than that of age without statistical significance (Z = 1.798, *p* = 0.0722). Our study indicated that AGR exhibited better performance than to initial GCS score in predicting GIB in patients with ICH. An interesting finding was that a significant difference was witnessed between the ICH location and GIB after PSM, although a statistically significant difference was not detected before PSM. This finding seemed inconsistent with prior studies (6, 7, 9, 26). The different results may be related to the different classifications of ICH locations. Further studies are needed to determine the association between ICH location and the occurrence of GIB. Another finding was that AGR levels were statistically correlated with unfunctional 90-day outcomes.

Our study has the following limitations. First, this was a single-center retrospective observational study, and multicenter prospective studies should corroborate the findings. Second, the timing of diagnosis and treatment of GIB after ICH onset was not controlled, but it was determined by each attending physician, which might affect the outcome. Third, the alignment of GCS scores between different physicians may impact this study's results. As a retrospective study, however, we could not evaluate this difference precisely. Fourth, the source of GIB could not be determined due to the inability to perform an endoscopy. Fifth, drugs such as glucocorticoids and antibiotics may have affected the results, but we did not assess their effects. Finally, our study did not include the National Institutes of Health Stroke Scale, which measures neurological impairment after stroke, due to the incompleteness of the records.

## Conclusion

Higher AGR was associated with an increased risk of GIB and unfunctional 90-day outcomes in patients with primary ICH. Nevertheless, we need further studies with large-sample, multicenter, and prospective clinical trials to validate our results.

## Data availability statement

The raw data supporting the conclusions of this article will be made available by the authors, without undue reservation.

## Ethics statement

The studies involving human participants were reviewed and approved by the Ethics Committee of the Second Affiliated Hospital of Fujian Medical University. The Ethics Committee waived the requirement of written informed consent for participation.

## Author contributions

WQ, CL, and HG designed the study and drafted the manuscript. WQ, CL, FY, and ZP collected and analyzed data. JY and GW helped in the statistical analysis and result interpretation. ZP, WQ, and WH prepared the figures and interpreted the results. HG and WH supervised the study and revised the manuscript, were identified as the guarantor of the article, and taking responsibility for the integrity of the study as a whole. All authors read and approved the final manuscript.

## References

[1] Cordonnier C Demchuk A Ziai W Anderson CS. Intracerebral haemorrhage: current approaches to acute management. Lancet. (2018) 392:1257–68. 10.1016/S0140-6736(18)31878-6 30319113

[2] Hanley DF Thompson RE Rosenblum M Yenokyan G Lane K McBee N. Efficacy and safety of minimally invasive surgery with thrombolysis in intracerebral haemorrhage evacuation (MISTIE III): a randomized, controlled, open-label, blinded endpoint phase 3 trial. Lancet. (2019) 393:1021–32. 10.1016/S0140-6736(19)30195-3 30739747 PMC6894906

[3] Zou Y Zhang W Huang C Zhu Y. Clinical significance of neutrophil to lymphocyte ratio and platelet to lymphocyte ratio in acute cerebral hemorrhage with gastrointestinal hemorrhage, and logistic regression analysis of risk factors. Exp Ther Med. (2019) 18:1533–8. 10.3892/etm.2019.7778 31410106 PMC6676203

[4] Wei J Jiang R Li L Kang D Gao G You C. Stress-related upper gastrointestinal bleeding in adult neurocritical care patients: a Chinese multicenter, retrospective study. Curr Med Res Opin. (2019) 35:181–7. 10.1080/03007995.2018.1448261 29499622

[5] Yang T-C Li J-G Shi H-M Yu D-M Shan K Li L-X. Gastrointestinal bleeding after intracerebral hemorrhage: a retrospective review of 808 cases. Am J Med Sci. (2013) 346:279–82. 10.1097/MAJ.0b013e318271a621 23221511

[6] Misra UK Kalita J Pandey S Mandal SK. Predictors of gastrointestinal bleeding in acute intracerebral haemorrhage. J Neurol Sci. (2003) 208:25–9. 10.1016/S0022-510X(02)00415-X 12639721

[7] Liu S Wang Y Gao B Peng J. A nomogram for individualized prediction of stress-related gastrointestinal bleeding in critically ill patients with primary intracerebral hemorrhage. Neuropsychiatr Dis Treat. (2022) 18:221–9. 10.2147/NDT.S342861 35177906 PMC8843804

[8] Liu BL Li B Zhang X Fei Z Hu SJ Lin W. A randomized controlled study comparing omeprazole and cimetidine for the prophylaxis of stress-related upper gastrointestinal bleeding in patients with intracerebral hemorrhage. J Neurosurg. (2013) 118:115–20. 10.3171/2012.9.JNS12170 23061387

[9] Granholm A Zeng L Dionne JC Perner A Marker S Krag M. Predictors of gastrointestinal bleeding in adult ICU patients: a systematic review and meta-analysis. Intensive Care Med. (2019) 45:1347–59. 10.1007/s00134-019-05751-6 31489445

[10] Fu J. Factors affecting the occurrence of gastrointestinal bleeding in acute ischemic stroke patients. Medicine. (2019) 98:28. 10.1097/MD.0000000000016312 31305417 PMC6641696

[11] Wang WJ Lu JJ Wang YJ Wang CX Wang YL Hoff K. Clinical characteristics, management, and functional outcomes in Chinese patients within the first year after intracerebral hemorrhage: analysis from China National Stroke Registry. CNS Neurosci Ther. (2012) 18:773–80. 10.1111/j.1755-5949.2012.00367.x 22943144 PMC6493640

[12] Feldman A Hart KW Lindsell CJ McMullan JT. Randomized controlled trial of a scoring aid to improve Glasgow Coma Scale scoring by emergency medical services providers. Ann Emerg Med. (2015) 65:325–329 e2. 10.1016/j.annemergmed.2014.07.454 25199613 PMC4339531

[13] Hemphill JC Greenberg SM Anderson CS Becker K Bendok BR Cushman M. Guidelines for the management of spontaneous intracerebral hemorrhage: a guideline for healthcare professionals from the american heart association/american stroke association. Stroke. (2015) 46:2032–60. 10.1161/STR.0000000000000069 26022637

[14] Chen H Guo Y Chen SW Wang G Cao HL Chen J. Progressive epidural hematoma in patients with head trauma: incidence, outcome, and risk factors, *Emerg Med Int*. (2012) 2012:134905. 10.1155/2012/134905 23320175 PMC3536037

[15] Von Haehling S Hartmann O Anker SD. Does obesity make it better or worse: insights into cardiovascular illnesses. Eur Heart J. (2013) 34:330–2. 10.1093/eurheartj/ehs237 22947611

[16] Kalogeropoulos AP Georgiopoulou VV Murphy RA Newman AB Bauer DC Harris TB. Dietary sodium content, mortality, and risk for cardiovascular events in older adults: the health, aging, and body composition (Health ABC) Study. JAMA Intern Med. (2015) 175:410–9. 10.1001/jamainternmed.2014.6278 25599120 PMC4785822

[17] Sarfo FS Ovbiagele B Gebregziabher M Akpa O Akpalu A Wahab K. Unraveling the risk factors for spontaneous intracerebral hemorrhage among West Africans. Neurology. (2020) 94:e998–e1012. 10.1212/WNL.0000000000009056 32075893 PMC7238923

[18] Wang CY Zhang YB Wang JQ Zhang XT Pan ZM Chen LX. Association between serum lactate dehydrogenase level and hematoma expansion in patients with primary intracerebral hemorrhage: a propensity-matched analysis. World Neurosurg. (2022) 160:e579–e590. 10.1016/j.wneu.2022.01.080 35093580

[19] Brouwers HB Chang Y Falcone GJ Cai X Ayres AM Battey TW. Predicting hematoma expansion after primary intracerebral hemorrhage. JAMA Neurol. (2014) 71:158–64. 10.1001/jamaneurol.2013.5433 24366060 PMC4131760

[20] Cook D Guyatt G. Prophylaxis against upper gastrointestinal bleeding in hospitalized patients. N Engl J Med. (2018) 378:2506–16. 10.1056/NEJMra1605507 29949497

[21] Pan Z Zhong Q Wang C Wang J Chen X Li X. Association between partial pressure of carbon dioxide and immediate seizures in patients with primary intracerebral hemorrhage: a propensity-matched analysis. Front Neurol. (2022) 13:865207. 10.3389/fneur.2022.865207 35528742 PMC9069159

[22] Tang CQ Matsui T Ohashi H Dong YF Momohara A Herrando-Moraira S. Identifying long-term stable refugia for relict plant species in East Asia. Nat Commun. (2018) 9:4488. 10.1038/s41467-018-06837-3 30367062 PMC6203703

[23] Zullo A Hassan C Campo SMA Morini S. Bleeding peptic ulcer in the elderly risk factors and prevention strategies. Drugs Aging. (2007) 24:815–28. 10.2165/00002512-200724100-00003 17896831

[24] ChenCMHsuHCYWCChuangCHLinCHHongCZ. Study on factorS affecting the occurrence of upper gaStrointeStinal bleeding in elderly acute stroke patients undergoing rehabilitation. J Nutr Health Aging. (2011) 15:632–6. 10.1007/s12603-011-0052-221968857

[25] Viderman D Issanov A Temirov T Goligher E la Fleur P. Outcome predictors of stroke mortality in the neurocritical care unit. Front Neurol. (2020) 11:579733. 10.3389/fneur.2020.579733 33384652 PMC7769840

[26] Wijdicks EFM Fulgham JR Batts KP. Gastrointestinal bleeding in stroke. Stroke. (1994) 25:2146–8. 10.1161/01.STR.25.11.2146 7974536

[27] Schaller BJ Graf R Jacobs AH. Pathophysiological changes of the gastrointestinal tract in ischemic stroke. Am J Gastroenterol. (2006) 101:1655–65. 10.1111/j.1572-0241.2006.00540.x 16863574

[28] Meyer JS. Stoica E, Pascu I, Hartmann A. Catecholamine concentrations in CSF and plasma of patients with cerebral infarction and haemorrhage. Brain. (1973) 96:277–88. 10.1093/brain/96.2.277 4715186

[29] Salottolo K Levy AS Slone DS Mains CW. Bar-Or D. The effect of age on Glasgow Coma Scale score in patients with traumatic brain injury. JAMA Surg. (2014) 149:727–34. 10.1001/jamasurg.2014.13 24899145

[30] Misra UK Kalita J Pandey S Mandal SK. Srivastava M. A randomized placebo controlled trial of ranitidine versus sucralfate in patients with spontaneous intracerebral hemorrhage for prevention of gastric hemorrhage. J Neurol Sci. (2005) 239:5–10. 10.1016/j.jns.2005.07.011 16182311

[31] Di Napoli M Parry-Jones AR Smith CJ Hopkins SJ Slevin M Masotti L. C-reactive protein predicts hematoma growth in intracerebral hemorrhage. Stroke. (2014) 45:59–65. 10.1161/STROKEAHA.113.001721 24262327

